# Optofluidic Flow Cytometer with In-Plane Spherical Mirror for Signal Enhancement

**DOI:** 10.3390/s23229191

**Published:** 2023-11-15

**Authors:** Filippo Zorzi, Silvio Bonfadini, Ludovico Aloisio, Matteo Moschetta, Filippo Storti, Francesco Simoni, Guglielmo Lanzani, Luigino Criante

**Affiliations:** 1 Center for Nano Science and Technology, Istituto Italiano di Tecnologia, Via Rubattino 81, 20134 Milan, Italy; filippo.zorzi@iit.it (F.Z.); silvio.bonfadini@iit.it (S.B.); ludovico.aloisio@iit.it (L.A.); matteo.moschetta@iit.it (M.M.); filippo.storti15@gmail.com (F.S.); guglielmo.lanzani@iit.it (G.L.); 2 Department of Physics, Politecnico di Milano, Piazza Leonardo da Vinci, 32, 20133 Milan, Italy; 3 Università Politecnica delle Marche, 60131 Ancona, Italy; f.simoni@photomat.it; 4 Institute of Applied Sciences and Intelligent Systems, Consiglio Nazionale delle Ricerche (CNR), 80072 Pozzuoli, Italy

**Keywords:** flow cytometry, optofluidic particles detection, FLICE, Lab on a Chip, femtosecond laser microfabrication

## Abstract

Statistical analysis of the properties of single microparticles, such as cells, bacteria or plastic slivers, has attracted increasing interest in recent years. In this regard, field flow cytometry is considered the gold standard technique, but commercially available instruments are bulky, expensive, and not suitable for use in point-of-care (PoC) testing. Microfluidic flow cytometers, on the other hand, are small, cheap and can be used for on-site analyses. However, in order to detect small particles, they require complex geometries and the aid of external optical components. To overcome these limitations, here, we present an opto-fluidic flow cytometer with an integrated 3D in-plane spherical mirror for enhanced optical signal collection. As a result, the signal-to-noise ratio is increased by a factor of six, enabling the detection of particle sizes down to 1.5 µm. The proposed optofluidic detection scheme enables the simultaneous collection of particle fluorescence and scattering using a single optical fiber, which is crucial to easily distinguishing particle populations with different optical properties. The devices have been fully characterized using fluorescent polystyrene beads of different sizes. As a proof of concept for potential real-world applications, signals from fluorescent HEK cells and *Escherichia coli* bacteria were analyzed.

## 1. Introduction

The ability to discern the composition of a fluid and, thus, the real-time monitoring of elements that may pose harm to humans are subjects of intense interest. Drinking water contaminated by bacteria, viruses and parasites, if not readily identified, can cause a range of health problems. In fact, it is widely recognized that diseases stemming from *Escherichia coli* (*E. coli*) in water cause hundreds of thousands of illnesses annually [1], and it is estimated to be the second leading cause of death in children under five years of age [2]. Therefore, any water analysis technique must be able to ensure compliance with strict quality standards for safe daily use and consumption. At the same time, the ability to analyze single cells up to large numbers within body fluids allows a wide range of scientific investigations to be carried out, including studying the effects of the same marker or pollutant on their physical and chemical properties. It has been shown that certain biological markers may be related to a number of different illnesses [3,4,5] or may indicate a predisposition to the development of future disease [6,7,8]. In addition, huge amounts of plastic are known to enter the oceans every year, and as they decompose, even very small debris (microplastics) can be harmful to wildlife [9] and humans [10]. To date, the effects on cells of ingesting these microplastics have not yet been studied in detail [11,12].

Flow cytometry is a commonly used, powerful and quantitative method for analyzing the properties of individual elements of a population mixed in a given fluid. It has a wide range of applications, including the diagnosis of blood cancer [13], DNA sequencing [14] and cell detection [15], to the extent that it is considered the gold standard for the statistical characterization of the biochemical and biophysical properties of individual cells/particles. It is complementary to imaging; although it does not provide the same level of cell detail, it offers several unique advantages, allowing for a multi-parametric, rapid and semi-quantitative analysis of cell populations (even heterogeneous ones) at the single cell level. Since the early days of cell counting, efforts have been made to automate the process to achieve high throughput while maintaining accuracy and providing user-friendly interfaces. On the market, we can find flow cytometers based on three operating principles: impedance analysis, image-based and optical-based cytometry. Because of the limitations of both electrochemical and mechanical techniques, optical detection is mostly preferred for its robustness and sensitivity [16,17,18]. In this case, the fluid to be analyzed (and the particles it contains) is placed in a specific area of the instrument and illuminated by a focused laser beam. The fluorescence and scattered light are collected at specific discrete angles and sent to detectors, which convert it into electrical signals for the analysis. 

The amplitude and shape of these electrical signals provide important information about the particle and/or cell. Scattering, due to light deflected from the original direction of propagation of the incident light, can be divided into forward scattering (FSC) and side scattering (SSC), and it provides information about the structure and morphology of the particle. In particular, FSC (low angle of beam deflection) is typically related to the size of the particle, although in a very approximate way, as the relationship proves to be unfortunately not monotonic for every application, while SSC (high angle of beam deflection) mainly contains information about the granularity/morphology and internal complexity of the micro-object/cell [19]. Fluorescence is proportional to the amount of fluorophore that enters or attaches externally to the particle, and it can be used to distinguish between different cell populations [20] and even to identify dead or membrane-deficient cells [21]. However, although there are currently instruments capable of analyzing up to 14 parameters simultaneously [22], commercial flow cytometers present several disadvantages: (i) misalignment of the small focal point of the probing laser with respect to the flowing micrometric object during the run time (even with high-performance optics) and (ii) a lack of flexibility in rearranging the measurement chamber, which can affect the analysis [18]. Additionally, these laboratory instruments are expensive (the cost can be of the order of tens of thousands of dollars), complex to use, bulky, handle relatively large (mL) sample volumes and require trained operators for operating and maintenance [23]. These factors make commercial flow cytometers difficult to use outside laboratories and strongly limit their use in remote and resource-poor areas, making them unsuitable for developing countries or for portable point-of-care diagnostics (PoC) [24,25].

To overcome all these problems, significant efforts have been made to miniaturize benchtop flow cytometers into microfluidic systems. Besides miniaturization, a microfluidic platform typically offers integration, automation and parallelization of (bio)chemical processes. As a result, microfluidic flow cytometers have many advantages, the most typical being low fluid consumption (μL-nL), high throughput, improved process control, increased sensitivity and compactness by integrating many functionalities in a single Lab on a Chip (LoC) of a few mm^2^. This pushes forward their portability, making them suitable for PoC testing even in underserved areas [26,27,28,29]. The reduced analysis volume in a miniaturized system leads to detection problems due to the consequently very low number of analytes available for a useful detection signal. For this reason, optical detection may also be the most suitable analysis method in a microfluidic chip due to its very high sensitivity and remarkable ease of integration with other functional units of an analytical device. By exploiting the higher efficiency of light–fluid interactions, the optofluidic platform represents an innovative way to improve the performance of sensing applications. In many opto-microfluidic cytometer designs, micro-optic components (integrated waveguides, lenses and fiber optics) are used to precisely manage excitation light and collect both scattered and fluorescence signals in a controlled way [18]. There are many solutions in the literature for placing a high-performance detection system directly on an optofluidic chip. Some of them focus on the implementation of integrated waveguides; although they can be precisely defined by the micromachining process, they tend to be thin in the vertical direction due to the limitations of the fabrication techniques and may not match the height of the buried fluidic channel to be interrogated. In addition, they typically have an attenuation at least five orders of magnitude larger than that of optical fibers [30]. 

The use of micro lenses, on the other hand, helps to increase the intensity of the pump signal and can facilitate the detection of light from the sample to the collecting optical fibers, substantially improving the efficiency of the measurements. Unfortunately, this makes the detection system more sensitive to misalignments between the area of investigation (micrometric focal point) and the hydrodynamic position of the sample caused by vibrations or non-optimal working conditions [18]. This aspect can be a major limitation as the sample size decreases, and it is essential to address this in order to have a device capable of making a reliable measurement. Some benefits can be gained by using external optics—such as objective lenses for better 3D control of the light spot—but their bulky size, susceptibility to shock and alignment would prevent the microfluidic system from being truly portable [31,32,33]. Instead, when multiple optical fibers are integrated into the device to increase the solid angle of detection of the fluorescence and scatter signal, many outputs (one per parameter) must be used, making the device fabrication and data analysis more complex [34].

Although each of the micro-optics’ contributions has important strengths, a proposal for a portable, easy-to-use device (no specialized staff required) that combines high sensitivity, high throughput and fast response is still lacking. Improving the accuracy of optofluidic flow cytometers and, therefore, the minimum detectable particle size at high throughput, two main critical factors need to be addressed: the precise control of the position in 3D of the microparticles in the flow and a high signal-to-noise ratio of measurement. To meet these requirements, we report here the fabrication and characterization of a new optofluidic flow cytometer that exploits the integration of an innovative and user-friendly 3D flow cell with an integrated in-plane 3D spherical micromirror, on the same glass platform, to increase S/N by several units. For smaller particle focusing, a new geometry [35] is designed to enable hydrodynamic focusing using only two inlets, exploiting a simple and fast manufacturing process. The same fabrication tool was used to simultaneously integrate the optical element into the chip, avoiding misalignments. The motivation for selecting this smart in-plane optical element is to ensure the collection of useful signals from a wider, non-discrete solid angle while reducing background noise by placing it as close as possible to the sensing core components. As a result, the obtained signal increase makes it possible to detect flowing particles down to a size of 1.5 microns (including bacteria) using only cheap photodetectors. The device was tested by individually identifying polystyrene beads of different sizes and morphology from their mixture, fluorescent HEK293T cells and the presence of bacteria in water. In our LoC, scattering and fluorescence are collected from the same fiber output; this simplifies the geometry and facilitates the processing of the two optical signals using simple and widely available integrated electro-optical tools. This makes the device robust and compact, with valuable advantages in terms of portability, sensitivity and ease of use.

## 2. Materials and Methods

### 2.1. Simulation

All the simulations presented in this work were carried out using the COMSOL software (COMSOL Multiphysics 5.3a). Different meshes were implemented, ultrafine for areas of major interest and looser for areas far from them. To simulate the trajectory of the light, a point source emitting 7000 spherical rays was chosen. To reduce the computation time, the number of secondary rays generated by reflections at the interfaces was set to zero, and the propagation losses inside the optical fiber were neglected.

### 2.2. Fabrication Process

To fabricate our optofluidic LoC, we used a femtosecond light induced chemical etching (FLICE) fabrication technique. It is a well-known direct-writing manufacturing technique for the rapid prototyping of 3D structures on a fused silica substrate [36,37,38,39,40,41,42]. In the first step, the glass substrate was irradiated with a femtosecond laser following a 3D path. Thanks to non-linear phenomena, it is possible to locally modify the properties of the material (only in the laser focal spot), thus realizing both the microfluidic circuit and the optical element scaffold in a single step. In fact, the 3D shape of the in-plane spherical mirror was achieved using the same fabrication tool. During the second step, the irradiated substrate is subjected to wet chemical etching to remove the material previously modified by the laser since it is etched faster than the non-written parts. This selectivity allows 3D geometries to be buried into a fused silica substrate without the need for post-processing (sealing, solving leakage problems, etc.).

Femtosecond laser irradiation was performed using the second harmonic of an ultrashort pulse laser (PHAROS PH2-10W, LightConversion, Vilnius, Lithuania). To exploit intensity-driven non-linear effects in the fs pulse regime, the beam was statically focused through an objective lens (50X M Plano APO NIR, Mitutoyo, Kanagawa, Japan), obtaining pulses of 420 nJ. To realize 3D trajectories, the substrate (fused silica, FOCKtech, Fujian, China) was placed on a 3-axis air-bearing stage (Fiber-Clide3D, Aerotech, Pittsburgh, Canada) controlled by CAD-based software (SCA v2.6.91, Altechna, Vilnius, Lithuania). The translation stage speed was set at 1 mm/s, while the beam polarization was set as orthogonal to the writing direction to ensure the maximum etching rate [43]. 

The subsequent chemical etching was carried out using two different etchants. The substrate was first immersed in an aqueous solution of 20% HF at 35 °C in an ultrasonic bath to remove the more external parts, while for the high spatial resolution part, it was immersed in an aqueous solution of 10 mM KOH at 95 °C again with ultrasonic application. Due to its better selectivity [44,45,46], KOH allows for more precise control of the size of the fiber housing and maintenance of the micromirror shape. 

Despite the ability of FLICE to produce 3D structures with micrometric precision, a residual surface roughness (RMS of the order of hundreds of nm) remains after chemical etching [47], which severely affects the reflective function of the mirror. Therefore, in order to improve the optical reflectivity, optical polishing was performed using a CO_2_ laser, as described by Storti et al. [48]. In this way, a final roughness below 5 nm was achieved, giving an optical quality to the surface. Finally, the curved surface of the scaffolding needed to be used as a spherical mirror was coated with a metal-based ink [48,49] (silver nanoparticles, ANP SilverJet DGP 40LT-15C) with the aim of increasing the reflectance up to 95%. To obtain a homogenous film, the ink was deposited by means of a DMATIX printer (DIMATIX DMP-2831, Fujifilm, Tokyo, Japan) and sintered on a hot plate at 150 °C for 5 min. Once the process is completed, the curved metallic mirror is ready to reflect and focus the signal into the collector fiber, which, thanks to the fabrication technique, is well aligned to all the other components of the optofluidic sensor. 

Finally, with the aim of connecting the microfluidic chip to the macroscopic world, tubes (in PEEK material) were placed in special horizontal accesses and sealed with a UV glue (NOA 63, Thorlabs, Newton, NJ, USA). Two optical fibers – a single-mode fiber (P1-460B-FC-2, Thorlabs, Newton, NJ, USA) for the excitation and a multimode one (M44L02, Thorlabs, Newton, NJ, USA) for the signal collection – were also placed and sealed in the designed accesses. 

### 2.3. Device Characterization Setup

The characterization of the chip’s sensing capabilities was performed by connecting the microfluidic platform to a controlled, constant-pressure injection system (OB1MK3, Elveflow, Paris, France), which ensures high resolution and stable pressure adjustment. The excitation fiber was connected to a 488 nm wavelength CW laser (ACX-HTSK-LBX, Oxxius, Paris, France).

**Figure 1:**
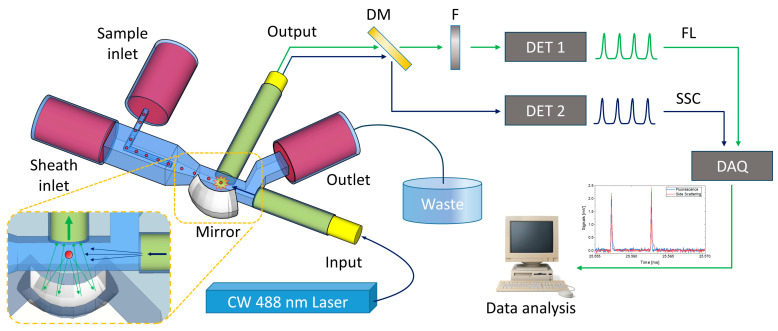
Sketch of the optofluidic chip and the detection apparatus. Once placed in the center of the outer channel via hydrodynamic focusing, the sample is excited, and the emitted light is collected by an optical fiber. The signal is divided into two spectral regions by a dichroic mirror (DM). A notch filter (F) was used in order to cut the pump beam in the fluorescent branch. The signals are converted by two photodetectors (DET 1 and 2), acquired by a DAQ and finally processed by NI LabVIEW 2017 software that provides a real-time analysis of the collected data.

As schematically shown in Figure 1, the separation of the scattered signal (in our case, at λ = 488 nm, although this scattering branch correctly receives wavelength < 490 nm) from the fluorescence signal (wavelength > 505 nm) is achieved using a kinematic multimode fiber optic filter cube (FOFD3/M-A, Thorlabs, Newton, NJ, USA), on which a proper dichroic mirror (MD498, Thorlabs, Newton, NJ, USA) is mounted. The output signals reach the two respective high-speed photodetectors (DET025AFC-M, Thorlabs, Newton, NJ, USA), which convert optical signals to electrical ones. A notch filter (NF488-15, Thorlabs, Newton, NJ, USA) was inserted along the fluorescent detection branch to eliminate any noise due to the excitation beam.

To convert the photocurrent generated by the photodiode into an output voltage, a load resistance was added at the connection between the photodiode and the Data Acquisition Card (DAQ). Different resistor values can be set, depending on the measurements, to provide the best trade-off between the electronic system bandwidth and absolute channel voltage response. Voltage signals are acquired simultaneously at 30 kHz by a 16-bit DAQ, (National Instruments, Austin, TX, USA) and analyzed by a customized LabVIEW interface. 

During the microfluidic tests, the chip was placed on the plate of an optical microscope (BX53M, Olympus, Tokyo, Japan) equipped with a high-speed camera (FASTCAM Mini UX100 type 800k-M-16G, Photron, Tokyo, Japan) to further validate the correct operation of the device.

### 2.4. Samples Preparation

In order to investigate the detection performance of the device, tests were performed using fluorescent microspheres and micro-organisms with a fluorescent dopant. Aqueous solutions with random concentrations of beads with a diameter of 10 µm (RedFluo PS Microspheres, EPRUI Biotech Co., Ltd., Shanghai, China), 6 µm (Alfa Aesar, 44,144 Polystyrene latex microspheres, fluorescent), 3.5 µm (PS-FluoGreen-3.5, Microparticles GmbH, Berlin, Germany), 1.5 µm PS-FluoGreen-1.5, Microparticles GmbH, Berlin, Germany) and a mixture of these were used for the microsphere tests. For the biological proof of concept, human embryonic kidney cells (HEK 293T purchased from ATCC) and bacteria from the 25,922 strain of *Escherichia coli* were used, made fluorescent using CellMask^TM^ or fluorescein diacetate (FDA) dyes.

HEK 293T cell preparation. Cells were cultured in T-25 cell culture flasks containing Dulbecco’s Modified Eagle Medium high-glucose (DMEM-HG) culture medium, supplemented with 10% heat-inactivated Fetal Bovine Serum (FBS) and 1% GlutaMAX (0.5 mM, Thermo Fisher Scientific, Waltham, MA, USA). Culture flasks were kept in a humidified incubator at 37 °C with 5% CO_2_. Before reaching 80% confluence, the cells were enzymatically detached from the flasks using a 1× trypsin-EDTA solution, centrifuged (5 min at 1200 rpm) and resuspended in 1 mL of DMEM. The cell suspension was then filtered through a PVDF 40 µm filter to prevent the microfluidic system from clogging. Finally, 2 µL of CellMask^TM^ Green (Thermo Fisher Scientific, Waltham, MA, USA) was incubated with the HEK293T cells for 5 min to stain their membranes and make them fluorescent. To remove unbound CellMask molecules, cells were centrifuged again (5 min at 1200 rpm), and the supernatant solution was taken out before resuspending cells in PBS.

*Escherichia coli* preparation. Luria–Bertani (LB) broth was used for liquid cultures. The liquid bacterial cultures were grown overnight in LB medium in an incubator at a constant temperature of 37 °C with a shaking speed of 200 rpm. Before the experiments, they were diluted to OD600 0.5. After treatment with 5 μg/mL of FDA for 5 min to make them fluorescent, the bacteria were suspended in water at random concentrations.

## 3. Results

### 3.1. Device Design and Simulations

**Figure 2:**
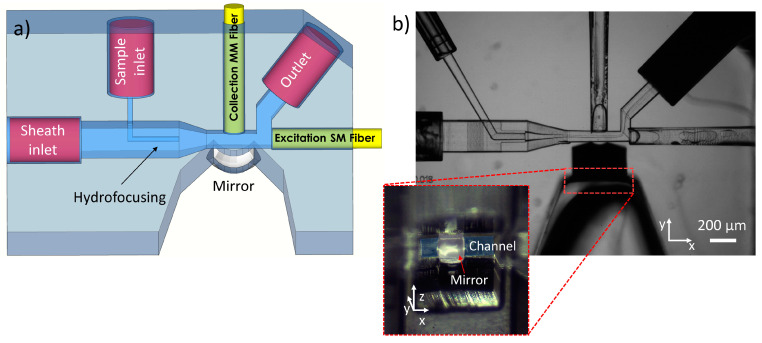
(**a**) Sketch of the optofluidic chip geometry. (**b**) Bright-field microscope image of the fabricated chip acquired with 5× objective lens. The image was acquired during the hydrodynamic focusing tests performed by pumping isopropanol inside the focusing channel and water in the sheath inlet. Zoom—side view of the mirror before the metallization.

In optical flow cytometry, it is crucial to have precise control over the position of the samples inside the microchannels; otherwise, the detection system loses reliability. For this reason, as shown in Figure 2, a first hydrodynamic focusing stage was added to the microfluidic detection chip. Taking advantage of the FLICE technique, a simplified version of the 3D hydrodynamic focusing proposed by Storti et al. [35] was carried out; it consists of only two inlets, one nested inside the other. The buffer flow acts as a sheath confining the sample stream, and all particles within it move to the center of the main channel where detection takes place. Moreover, it is possible to control the size of the focused flow by changing, in real-time, the pressures of the inlet channels (sample and sheath). Aiming to define the working point for the experiments, the device was tested by pumping water into the sheath channel and isopropyl alcohol inside the focusing channel. Thanks to their different refractive indexes, it was possible to directly observe the interface between the two fluids. The minimum size of the focused flow obtained was 3.5 µm (Figure 2b).

The detection stage was made up of an excitation source and a detection array. The excitation source was a 488 nm CW laser coupled to a single-mode fiber directly integrated inside the chip. The signal collection system consists of a multimode fiber (50 µm core diameter) and an in-plane 3D optical microelement (spherical mirror) buried in the fused silica substrate, exploiting a combination of FLICE and inkjet printing. The particles to be detected move towards the center of the channel thanks to hydrodynamic focusing and are illuminated by the excitation fiber; only when they approach the investigation area is their emitted light collected and focused into the fiber by the curved mirror (magnification in Figure 1). 

The 90-degree configuration between the excitation source and the collection fiber leads to a double benefit: on one hand, it prevents the transmitted pump signal from directly entering the collection system (as would be the case, in particular, with transparent particles such as cells) and overwhelming the optical signals that are useful for the measurement, thus strongly reducing the signal-to-noise ratio (S/R) [50]; on the other hand, it simplifies the collection geometry with a single common fiber for both fluorescence (FLUO) and side scattering (SSC) signals. 

**Figure 3:**
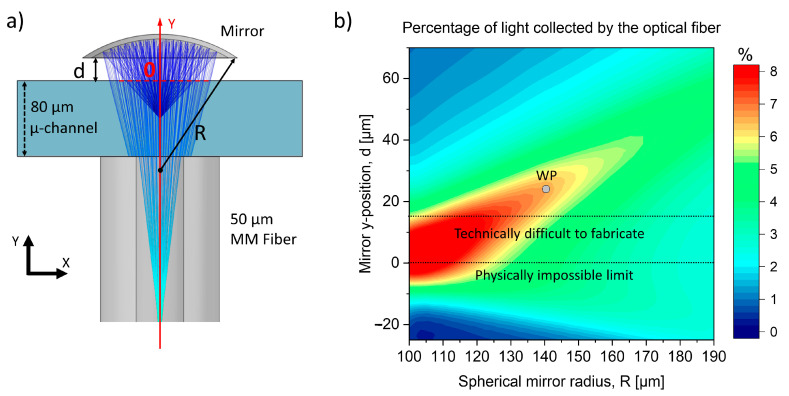
(**a**) Sketch of the geometry used for the simulation made with COMSOL Multiphysics. (**b**) Results obtained from the simulations. The working point (WP) corresponds to the mirror with a radius of 140 µm and with its center located at 24 µm from the microchannel.

Optimization was performed using COMSOL Multiphysics (base and geometric optics module) in order to determine the radius (R) and position of the mirror relative to the microfluidic channel (d) that maximize the collection efficiency of the detection system (curved mirror + fiber optic) along the red line (see Figure 3a). A point source of spherical rays positioned at the center of the microchannel and on the axis of the fiber (−40 µm position in Figure 3a) was used to calculate the fraction of light collected by the fiber for each mirror position and radius. The resulting map reported in Figure 3b shows how reducing the curvature of the mirror (R < 170 µm) increases the light collected as the mirror approaches the microfluidic channel (d → 0 μm).

Positions (d) below zero are physically impossible because it would require the mirror to be placed inside the microfluidic channel. The range between 0 and 15 µm is very difficult to realize because of the thin glass wall that defines the microchannel and supports the spherical mirror. In this case, the chip would be too fragile, with a further risk of breakage due to the high temperatures during the optical polishing. For these reasons, this distance cannot be reduced below a defined margin. Balancing collection efficiency and reliability fabrication, a spherical mirror with radius of 140 µm (f = 70 µm) was realized, with its center located inside the collecting fiber at 24 µm from the second wall of the microchannel. In this way, the expected fraction of collected light is about 7% of the total emitted light, compared to the 0.68% expected without the in-plane optics. Accordingly, this mirror can guarantee 10-fold increase in signal collection.

### 3.2. Microfluidics Experiments

**Figure 4:**
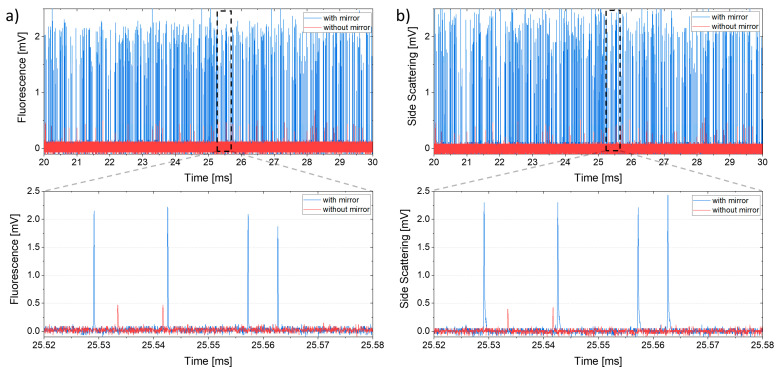
Comparison of fluorescence (**a**) and side scattering (**b**) signals from 3.5 µm beads obtained with (blue) and without (red) the integration of the spherical mirror.

Several experiments were carried out with the aim of highlighting the improvements obtained by integrating the spherical mirror into optofluidic flow cytometers. A first test was conducted by comparing two identical chips, one with the mirror and one without it. By keeping the same working conditions, (p_sample_ = 90 mbar; p_sheath_ = 100 mbar; P_laser_ = 20 mW; R_load_FLUO_ = 100 kΩ; R_load_SSC_ = 250 kΩ; sampling rate = 30 kHz) the signal produced by a 3.5 µm-diameter fluorescent polystyrene bead was acquired. As shown in Figure 4, the integration of the spherical mirror increases the average peak amplitude in both cases, FLUO and SSC, from 0.30–0.37 mV to 1.80–1.86 mV (variation coefficient about 0.5), with an average gain of about six times. Moreover, the noise affecting the measuring device seems to be independent of the presence or absence of the mirror, as can be seen from the zooms in Figure 4. Therefore, the actual S/N gain will be identical to the enhancement obtained with the useful signal (about six times in our case).

**Figure 5:**
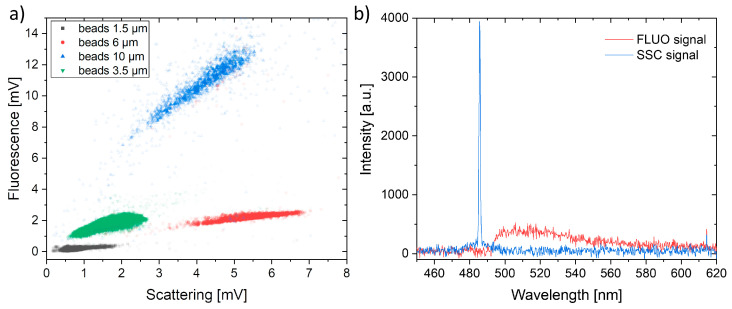
(**a**) Scattering and fluorescence signals obtained using a sample with a mix of fluorescent polystyrene spheres. (**b**) Spectral analysis of the light collected by the fibers after the signal splitting.

The proposed chip geometry enables the simultaneous (in time and space) enhancement and acquisition of both signals (fluorescence and scattering) on the same detection fiber. To characterize the detection capabilities of the chip as an optofluidic flow cytometer, the signals from a mixture of florescent beads of different sizes (with diameters of 10 µm, 6 µm, 3.5 µm and 1.5 µm) were compared. In particular, all the beads were functionalized with the same fluorophore, except the 6-micron family, which is loaded with a less efficient one. The combination of fluorescence and scattering information significantly contributes to the correct identification of the four beads populations. This is very clear from Figure 5a. For instance, by looking at the signals correspondent to the 3.5 and 6 µm beads populations, we observe that if only the fluorescence signal is considered (between 1 and 2 mV for both populations), they could not be distinguished, while they correspond to very different scattering signals (0.5–2.5 for 3.5 µm beads and 3.5–7.0 for 6 µm beads), thus allowing the different populations to be clearly identified. Similarly, the difference between the 1.5 and 3 µm beads is mainly in their fluorescence emission, while the two populations produce a very similar scattering signal.

Figure 5b shows the two typical spectrum acquired on the two different channels (fluorescence and scattering). As can be seen, there is no spectral overlap between the two signals, which ensures that the signals analyzed (simultaneously) in the two channels are due to the two different phenomena and, therefore, that the analysis is genuine. The detection setup, based on high-speed photodiodes with transimpedance and fast digital sampling cards (without video processing), allows for easy sampling and the acquisition of fast dynamic events down to microseconds (bandwidth of MHz). At the inlet pressures used in our study, the acquired peaks had a duration of about 240 µs, which is well below the bandwidth limits of the entire setup. By avoiding any kind of distortion (aliasing) in the digital signal processing, the analysis could be sped up (at least by a factor of 100). Nevertheless, the instrument performance in our specific case is that of a high-throughput flow cytometer capable of counting over 4000 particles per second. The proposed hydrodynamic focusing geometry is particularly suitable for the successful single-file 3D alignment and separation of particle clouds, regardless of their size. The resulting accurate control of the position of the flow particle has a positive effect on the signals’ stability and improves their collection. As a further advantage, the integration of the 3D spherical mirror allows small particles (1.5 µm) to be detected that would otherwise be difficult to obtain with confidence. 

**Figure 6:**
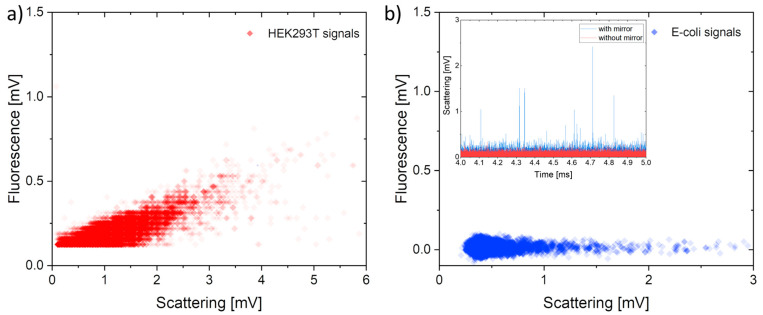
(**a**) Correlation between scattering and fluorescence signals obtained by analyzing HEK293T cells made fluorescent by CellMask^TM^ molecules. (**b**) Signals obtained by analyzing a water sample contaminated by *Escherichia coli* bacteria using the chip with a spherical mirror. In this case, the fluorescence signal was below the detection limit. Incept—time behavior of the *E. coli* scattering signal obtained with (blue) and without (red) the spherical mirror.

A further characterization of the device was carried out using biological samples to simulate a potential real-world application. In particular, breast adenocarcinoma cells (HEK293T) were used, made fluorescent with CellMask^TM^ (Thermo Fisher Scientific, Waltham, MA, USA) and suspended in fresh DMEM. The cells used have a suspension size in the range of 3 to 10 µm, while their fluorescence is 80 times smaller than that of 10 µm fluorescent microspheres. This claim was validated using a confocal microscope, which provides high sensitivity in fluorescence detection. Therefore, the load resistors were reversed (R_load_FLUO_ = 250 kΩ, R_load_SSC_ = 100 kΩ) to obtain a higher amplification of the fluorescence signal. The increase in load resistance may also affect the noise. In our case, however, it remains negligible (only tens of μV), making it possible to detect smaller peaks previously hidden by the noise oscillations (for more detailed information on how resistance change affects measurement, see Supplementary Materials). Figure 6a shows the cell distribution obtained with p_sample_ = 90 mbar, p_sheath_ = 105 mbar, P_laser_ = 20 mW and sampling rate = 30 kHz. As expected, the scattering signal of the cells is almost the same as that of the same-sized beads (taking into account the 2.5-fold ratio of the respective load resistance), while the fluorescent signal is about 32 times weaker than that of the 10 µm microspheres, which is in agreement with preliminary confocal microscope tests. 

Finally, a water sample contaminated by *Escherichia coli* bacteria was analyzed. Attempts were also made to make the bacteria fluorescent with FDA; unfortunately, the protocol used was not suitable to achieve a sufficient dopant concentration inside these micro-organisms. In fact, the confocal microscope detected fluorescence signals from bacteria at least an order of magnitude lower than those from the cells.

The working conditions were set as follows: p_sample_ = 70 mbar, p_sheath_ = 90 mbar, P_laser_ = 20 mW and R_load_SSC_ = 250 kΩ sampling rate = 30 kHz. As shown in Figure 6b, the device was able to detect the bacteria, although, as expected, only by scattering. The integration of the in-plane spherical micromirror was even more crucial in this case, as the inset clearly shows: without this micro-optics, no peak related to the passage of the bacterium can be identified. Unfortunately, the fluorescence signal was below the noise. However, by improving the dye doping protocol of bacteria and by exploiting the full bandwidth of the electronic signal acquisition system (increasing R_load_ to the maximum allowed), it is reasonable to foresee that a higher fluorescence “electric” signal could be achieved while maintaining a sufficient temporal resolution.

## 4. Discussion and Conclusions

Optofluidic flow cytometers are emerging in the LoC community as a key tool for many applications, from cell diagnostics to fluid analysis, allowing for the statistical characterization of the biochemical and biophysical properties of individual particles. 

Compared to the commercial laboratory version, which is expensive, bulky and not easy to use, the optofluidic version offers a simple, portable solution without compromising sensitivity or high throughput while providing valuable flexibility to integrate multiple components on the same platform, resulting in a powerful device that is cost-effective, automated and easy to use.

Exploiting these LoC capabilities, we proposed an optofluidic flow cytometer that integrates a unique 3D hydrodynamic focusing microfluidic cell with an in-plane 3D spherical micromirror to increase the signal-to-noise ratio, an improvement necessary for the correct detection of micrometric particles. The three-dimensional optical element captures the signal from a wider solid angle because it is curved and integrated in the device very close to the emission/diffusion source, focusing the reflected signals on the entrance of the single collection fiber. The special configuration also allows the scattering and fluorescence signals of the individual particle passing through the detection area to be collected simultaneously. The pump beam impinges at 90° with respect to the axis of the collection system, allowing for a strong depletion of noise associated with the ballistic transmitted signal. Additionally, this geometry makes the fabrication process simpler and more straightforward.

The devices’ performance was investigated through the following steps: simulation, implementation and characterization. The choice of mirror location and size was optimized according to results of simulations with COMSOL. 

It has been pointed out how the simultaneous acquisition of fluorescence and scattering signals—using only one optical fiber—is a key factor in easily distinguishing populations of particles with different optical properties, as in the case of the 3.5 and 6 µm polystyrene beads used. 

Proof of concept tests of the device were performed using HEK293T cells and *Escherichia coli* bacteria made fluorescent with dopant molecules. The integrated micromirror significantly increased the signal-to-noise ratio for both scattering and fluorescence when sufficiently observable. Regarding scattering—an optical property directly related to the geometric characteristics of the object—the device was able to easily detect micron-sized objects (down to 1.5 µm) and bacteria. Concerning fluorescence, the dye-doping protocol plays a key role in the light emission efficiency and therefore affects the detection capability. We are confident that by carefully selecting fluorescent molecules and engineering the chemical bond between them and the micrometric bio sample (as bacteria), it will be possible to obtain a fluorescence signal, overcoming the noise limit, thus allowing the device to be fully exploited for biodetection. In addition, since the device is made entirely of inert material (fused silica), it can be easily washed even with aggressive solvents that can completely remove all biological elements (e.g., chloroform, piranha solution) and therefore be reused many times. 

A remarkable feature of this device is its high throughput in detecting cells and bacteria—the latter impossible to detect without the integration of a spherical mirror—at over 4000 events per second without the need for expensive high-frame-rate vision systems or time-consuming post-processing analyses.

The possibilities offered by this innovative platform, in particular the easy integration of the optical element with microfluidic technology, help to bridge the gap between the macroscopic world and chip-based analyses, paving the way for automated, portable and high-throughput applications capable of detecting small particles below the micron-size while analyzing their different properties at the same time. The S/N enhancement achieved (over six times) can be seen as a first step towards using these devices for more challenging applications, such as the detection of microplastics in water samples or the detection of circulating tumor cells in biological samples.

## Data Availability

The data that support the findings of this study are available from the corresponding author upon reasonable request.

## Supplementary Materials

The following supporting information can be downloaded at: https://www.mdpi.com/article/10.3390/s23229191/s1, Figure S1: (a) Time behavior of the HEK293T cells (blue—side scattering and red—fluorescence) made fluorescent by CellMask molecules. Operating condition: R_load_FLUO_ = 250 kΩ, R_load_SSC_ = 100 kΩ, p_sample_ = 90 mbar, p_sheath_ = 105 mbar, P_laser_ = 20 mW and sampling rate= 30 kHz. (b) Schematization of how increased resistance affects detector and DAQ noises.

## References

[1] Rangel J.M., Sparling P.H., Crowe C., Griffin P.M., Swerdlow D.L. (2005). Epidemiology of *Escherichia Coli* O157:H7 Outbreaks, United States, 1982–2002. Emerg. Infect. Dis..

[2] World Health Organization, UNICEF (2013). Ending Preventable Child Deaths from Pneumonia and Diarrhoea by 2025: The Integrated Global Action Plan for Pneumonia and Diarrhoea (GAPPD).

[3] Ankeny J.S., Court C.M., Hou S., Li Q., Song M., Wu D., Chen J.F., Lee T., Lin M., Sho S. (2016). Circulating Tumour Cells as a Biomarker for Diagnosis and Staging in Pancreatic Cancer. Br. J. Cancer.

[4] Leng S.X., McElhaney J.E., Walston J.D., Xie D., Fedarko N.S., Kuchel G.A. (2008). ELISA and Multiplex Technologies for Cytokine Measurement in Inflammation and Aging Research. J. Gerontol. A Biol. Sci. Med. Sci..

[5] Yang S.-Y., Lien K.-Y., Huang K.-J., Lei H.-Y., Lee G.-B. (2008). Micro Flow Cytometry Utilizing a Magnetic Bead-Based Immunoassay for Rapid Virus Detection. Biosens. Bioelectron..

[6] Keating N.L., Pace L.E. (2018). Breast Cancer Screening in 2018. JAMA.

[7] Mincarelli L., Lister A., Lipscombe J., Macaulay I.C. (2018). Defining Cell Identity with Single-Cell Omics. Proteomics.

[8] Strzelecka P.M., Ranzoni A.M., Cvejic A. (2018). Dissecting Human Disease with Single-Cell Omics: Application in Model Systems and in the Clinic. Dis. Model. Mech..

[9] Jambeck J.R., Geyer R., Wilcox C., Siegler T.R., Perryman M., Andrady A., Narayan R., Law K.L. (2015). Plastic Waste Inputs from Land into the Ocean. Science.

[10] Gambino I., Bagordo F., Grassi T., Panico A., De Donno A. (2022). Occurrence of Microplastics in Tap and Bottled Water: Current Knowledge. Int. J. Environ. Res. Public Health.

[11] Campanale C., Massarelli C., Savino I., Locaputo V., Uricchio V.F. (2020). A Detailed Review Study on Potential Effects of Microplastics and Additives of Concern on Human Health. Int. J. Environ. Res. Public Health.

[12] Lim X. (2021). Microplastics Are Everywhere—but Are They Harmful?. Nature.

[13] Hasegawa D., Bugarin C., Giordan M., Bresolin S., Longoni D., Micalizzi C., Ramenghi U., Bertaina A., Basso G., Locatelli F. (2013). Validation of Flow Cytometric Phospho-STAT5 as a Diagnostic Tool for Juvenile Myelomonocytic Leukemia. Blood Cancer J..

[14] Sandberg J., Werne B., Dessing M., Lundeberg J. (2011). Rapid Flow-Sorting to Simultaneously Resolve Multiplex Massively Parallel Sequencing Products. Sci. Rep..

[15] Gross H.-J., Verwer B., Houck D., Recktenwald D. (1993). Detection of Rare Cells at a Frequency of One per Million by Flow Cytometry. Cytometry.

[16] Rusling J.F., Kumar C.V., Gutkind J.S., Patel V. (2010). Measurement of Biomarker Proteins for Point-of-Care Early Detection and Monitoring of Cancer. Analyst.

[17] Foudeh A.M., Fatanat Didar T., Veres T., Tabrizian M. (2012). Microfluidic Designs and Techniques Using Lab-on-a-Chip Devices for Pathogen Detection for Point-of-Care Diagnostics. Lab Chip.

[18] Yang H., Gijs M.A.M. (2018). Micro-Optics for Microfluidic Analytical Applications. Chem. Soc. Rev..

[19] Shapiro H.M. (2003). Practical Flow Cytometry.

[20] Macey M.G., Macey M.G. (2007). Principles of Flow Cytometry. Flow Cytometry: Principles and Applications.

[21] Rosenauer M., Buchegger W., Finoulst I., Verhaert P., Vellekoop M. (2011). Miniaturized Flow Cytometer with 3D Hydrodynamic Particle Focusing and Integrated Optical Elements Applying Silicon Photodiodes. Microfluid. Nanofluidics.

[22] Wilkerson M.J. (2012). Principles and Applications of Flow Cytometry and Cell Sorting in Companion Animal Medicine. Vet. Clin. N. Am. Small Anim. Pract..

[23] Melamed M.R. (2001). Chapter 1 A Brief History of Flow Cytometry and Sorting. Methods in Cell Biology.

[24] Gwimbi P., George M., Ramphalile M. (2019). Bacterial Contamination of Drinking Water Sources in Rural Villages of Mohale Basin, Lesotho: Exposures through Neighbourhood Sanitation and Hygiene Practices. Environ. Health Prev. Med..

[25] Haselgrübler T., Haider M., Ji B., Juhasz K., Sonnleitner A., Balogi Z., Hesse J. (2014). High-Throughput, Multiparameter Analysis of Single Cells. Anal. Bioanal. Chem..

[26] Rajawat A., Tripathi S. (2020). Disease Diagnostics Using Hydrodynamic Flow Focusing in Microfluidic Devices: Beyond Flow Cytometry. Biomed. Eng. Lett..

[27] Whitesides G.M. (2006). The Origins and the Future of Microfluidics. Nature.

[28] Niculescu A.-G., Chircov C., Bîrcă A.C., Grumezescu A.M. (2021). Fabrication and Applications of Microfluidic Devices: A Review. Int. J. Mol. Sci..

[29] Nahavandi S., Baratchi S., Soffe R., Tang S.-Y., Nahavandi S., Mitchell A., Khoshmanesh K. (2014). Microfluidic Platforms for Biomarker Analysis. Lab Chip.

[30] Ateya D.A., Erickson J.S., Howell P.B., Hilliard L.R., Golden J.P., Ligler F.S. (2008). The Good, the Bad, and the Tiny: A Review of Microflow Cytometry. Anal. Bioanal. Chem..

[31] Chen Y., Nawaz A.A., Zhao Y., Huang P.-H., McCoy J.P., Levine S.J., Wang L., Huang T.J. (2014). Standing Surface Acoustic Wave (SSAW)-Based Microfluidic Cytometer. Lab Chip.

[32] Grenvall C., Antfolk C., Bisgaard C.Z., Laurell T. (2014). Two-Dimensional Acoustic Particle Focusing Enables Sheathless Chip Coulter Counter with Planar Electrode Configuration. Lab Chip.

[33] Mao X., Lin S.-C.S., Dong C., Huang T.J. (2009). Single-Layer Planar on-Chip Flow Cytometer Using Microfluidic Drifting Based Three-Dimensional (3D) Hydrodynamic Focusing. Lab Chip.

[34] Mohan A., Gupta P., Nair A.P., Prabhakar A., Saiyed T. (2020). A Microfluidic Flow Analyzer with Integrated Lensed Optical Fibers. Biomicrofluidics.

[35] Storti F., Bonfadini S., Criante L. (2023). Simplified 3D Hydrodynamic Flow Focusing for Lab-on-Chip Single Particle Study. Sci. Rep..

[36] Martínez Vázquez R., Bragheri F., Paiè P. (2022). New Trends and Applications in Femtosecond Laser Micromachining.

[37] Gattass R.R., Mazur E. (2008). Femtosecond Laser Micromachining in Transparent Materials. Nat. Photonics.

[38] Sugioka K., Cheng Y. (2012). Femtosecond Laser Processing for Optofluidic Fabrication. Lab Chip.

[39] Qiu J., Miura K., Hirao K. (2008). Femtosecond Laser-Induced Microfeatures in Glasses and Their Applications. J. Non-Cryst. Solids.

[40] Marcinkevičius A., Juodkazis S., Watanabe M., Miwa M., Matsuo S., Misawa H., Nishii J. (2001). Femtosecond Laser-Assisted Three-Dimensional Microfabrication in Silica. Opt. Lett..

[41] Osellame R., Cerullo G., Ramponi R. (2012). Femtosecond Laser Micromachining: Photonic and Microfluidic Devices in Transparent Materials. Topics in Applied Physics.

[42] Hnatovsky C., Taylor R.S., Simova E., Rajeev P.P., Rayner D.M., Bhardwaj V.R., Corkum P.B. (2006). Fabrication of Microchannels in Glass Using Focused Femtosecond Laser Radiation and Selective Chemical Etching. Appl. Phys. A.

[43] Hnatovsky C., Taylor R.S., Simova E., Bhardwaj V.R., Rayner D.M., Corkum P.B. (2005). Polarization-Selective Etching in Femtosecond Laser-Assisted Microfluidic Channel Fabrication in Fused Silica. Opt. Lett..

[44] Gottmann J., Hermans M., Ortmann J. (2013). Microcutting and Hollow 3d Microstructures in Glasses by In-Volume Selective Laser-Induced Etching (Isle). J. Laser Micro Nanoeng..

[45] Hermans M., Gottmann J., Riedel F. (2014). Selective, Laser-Induced Etching of Fused Silica at High Scan-Speeds Using KOH. J. Laser Micro Nanoeng..

[46] Yashunin D.A., Malkov Y.A., Mochalov L.A., Stepanov A.N. (2015). Fabrication of Microchannels in Fused Silica Using Femtosecond Bessel Beams. J. Appl. Phys..

[47] Lo Turco S., Di Donato A., Criante L. (2017). Scattering Effects of Glass-Embedded Microstructures by Roughness Controlled Fs-Laser Micromachining. J. Micromech. Microeng..

[48] Storti F., Bonfadini S., Di Donato A., Criante L. (2022). 3D In-Plane Integrated Micro Reflectors Enhancing Signal Capture in Lab on a Chip Applications. Opt. Express.

[49] Simoni F., Bonfadini S., Spegni P., Turco S.L., Lucchetta D.E., Criante L. (2016). Low Threshold Fabry-Perot Optofluidic Resonator Fabricated by Femtosecond Laser Micromachining. Opt. Express.

[50] Watts B.R., Zhang Z., Xu C.-Q., Cao X., Lin M. (2013). A Method for Detecting Forward Scattering Signals On-Chip with a Photonic-Microfluidic Integrated Device. Biomed. Opt. Express.

