# Supplementary material for: Optofluidic Flow Cytometer with In-Plane Spherical Mirror for Signal Enhancement
Source: Sensors (Basel). 2023 Nov 15;23(22):9191. doi: 10.3390/s23229191 (PMC10675696; doi:10.3390/s23229191)
Supplement: Supplementary file 1 [file sensors-23-09191-s001.zip › sensors-2659941-supplementary.pdf]

sensors
MDPI

Supporting Information

Optofluidic Flow Cytometer with In-Plane Spherical Mirror for Signal Enhancement

Filippo Zorzi [1,2], Silvio Bonfadini [1], Ludovico Aloisio [1,2], Matteo Moschetta [1], Filippo Storti [1], Francesco Simoni [3,4], Guglielmo Lanzani [1] and Luigino Criante [1,*]

1 Center for Nano Science and Technology, Istituto Italiano di Tecnologia, Via Rubattino 81, 20134 Milan, Italy; filippo.zorzi@iit.it (F.Z.); silvio.bonfadini@iit.it (S.B.); ludovico.aloisio@iit.it (L.A.); matteo.moschetta@iit.it (M.M.); filippo.storti15@gmail.com (F.S.); guglielmo.lanzani@iit.it (G.L.)
2 Department of Physics, Politecnico di Milano, Piazza Leonardo da Vinci, 32, 20133 Milano, Italy
3 Università Politecnica delle Marche, 60131 Ancona, Italy; f.simoni@photomat.it
4 Institute of Applied Sciences and Intelligent Systems, Consiglio Nazionale delle Ricerche (CNR), 80072 Pozzuoli, Italy
* Correspondence: luigino.criante@iit.it

1. Noise analysis

Noise affecting analysis in an optical flow cytometer can come from two different sources: electronic noise (due to the detection system) and optical noise. The detection noise can be categorized as thermal noise, shot noise, quantization noise and dark current noise. These types of noise are strictly correlated to the detection setup (Photodiode, DAQ). In scattering based studies (SSC and FSC), the optical noise is mainly due to the direct pump signal (the ballistic transmitted signal), which may accidentally reach the detector but does not contribute to the measurement. Under the same working conditions (i.e., keeping the load resistance equal), the noise of our devices (with and without mirror) is the same, as can be seen in the "zoom inset" of Figures 4a and b (Main text). This confirms that the only noise affecting our device is electronic noise (otherwise the mirror would have increased the background signal due to optical noise). The main reason lies in the peculiar 90° configuration between the pump and the sensing paths that has been implemented. This choice allows pump light to reach the detector only if there is a particle capable of scattering light, the amount of which depends on the particle's properties.

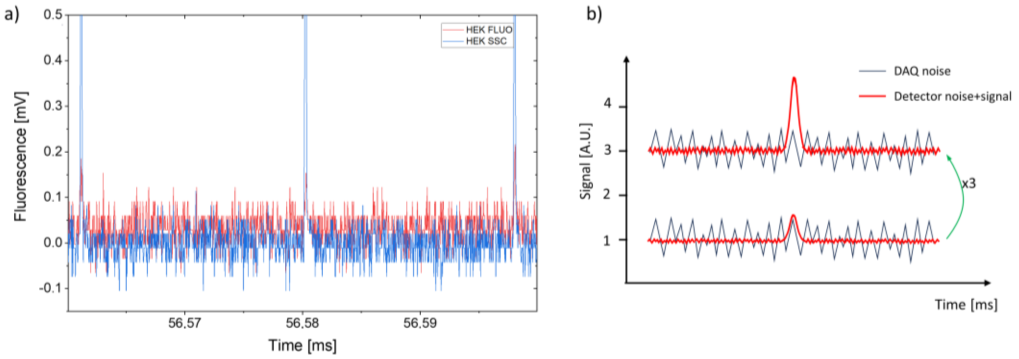

Figure S1. (a) Time behavior of the HEK293T cells (blue - side scattering - and red - fluorescence -) made fluorescent by CellMask molecules. Operating condition: R_{load_FLUO}= 250 kΩ, R_{load_SSC}= 100 kΩ, p_{sample}= 90 mbar, p_{sheath}= 105 mbar, P_{laser}= 20 mW and sampling rate= 30 kHz. (b) Schematization of how increased resistance affects detector and DAQ noises.

The electronic noise linked with our setup is mainly due to the components of the photodetector noise and DAQ noise. The increase in load resistance amplifies the signal and the noise from the detector but not the noise introduced by the DAQ. As can be seen from Figure S1.a, increasing the load resistance increases the noise baseline (average noise of red signal N_{red_mean}=0.0307 mV, with R_{load_FLUO}= 250 kΩ – $N_{blue\_ mean}$=0.0035 mV with R_{load_SSC}= 100 kΩ), while the noise oscillation remains the same (standard deviation of about 0.035 mV for both noises). This means that the contribution of this oscillation is to be attributed to noise from the DAQ and not to the detector and is therefore not amplified by the resistor change. On the other hand, the photodetector noise contribution is also amplified, but to an almost negligible value (only tens of μV) due to its low dark currents (ten of pA). In other words, the increase in resistance makes it possible to detect peaks that were previously hidden by the noise oscillation, as shown in the sketch of Figure S1.b.

When considering the maximum allowable value of the load resistance ($R_{L\ max}$), the response speed (bandwidth) of the detection system must also be considered. As the load resistance increases, the bandwidth of the photodetector's equivalent electronic circuit is reduced, making it impossible to acquire a fast signal. The bandwidth of a photodetector is calculated as

$$f_{BW} = \frac{1}{2\pi C_j R_L}$$

where C_j=1.73pF is the junction capacitance of the diode and R_L the load resistance. Since our typical events occur in about t=250 μs (@ p_{sample}=90 mbar, p_{sheath}=100 mbar), and f_{BW}=0.35/t_{rise} we can estimate the maximum load resistance as R_{load_max}=t/(1.4πC_j), obtaining a theoretical value of about 33MΩ.
